# Supplementary material for: The impact of climate change on ecology of tick associated with tick-borne diseases
Source: PLoS Comput Biol. 2025 Apr 8;21(4):e1012903. doi: 10.1371/journal.pcbi.1012903 (PMC12002643; doi:10.1371/journal.pcbi.1012903)
Supplement: S1 Text — (PDF) [file pcbi.1012903.s001.pdf]

# S1 Text. Estimation of parameters

## 1 Estimation of the climate-dependent parameters

In this study, we estimate the total 15 parameters(coefficients) $(a_i, i = 0, \cdots, 14)$ of climate-dependent parameters described in Table 1 using the *lsqcurvefit* in MATLAB. The least square method minimizes the sum of squares of residual between actual data (collected tick data) and simulated results by fitted parameter. The progress of the fitting development rate is divided into several steps.

1. Define the development rate and the reproduction rate, $d_1(T, H), d_2(T), d_3(T), d_4(T)$, as a function in Table 1.
2. Give initial conditions for the coefficients of each development rate and the reproduction rate function, $a_i, i = 0, \cdots, 14$.
3. Using the actual climate data (Temperature, Relative humidity) and initial condition, each development rate and reproduction rate at time $t$ is defined by development rate and reproduction rate functions.
4. Update the $a_i, i = 0, \cdots, 14$ using *lsqcurvefit* in MATLAB that minimizing following residual.

$$\text{Residual} = (d_1(T, H)(1 - N_V/K)E - C_L)^2 + (d_2(T)L - C_N)^2 + (d_3(T)N - C_A)^2,$$

where $C_L$ is the number of collected larvae, $C_N$ is the number of collected nymphs, and $C_A$ is the number of collected adults.

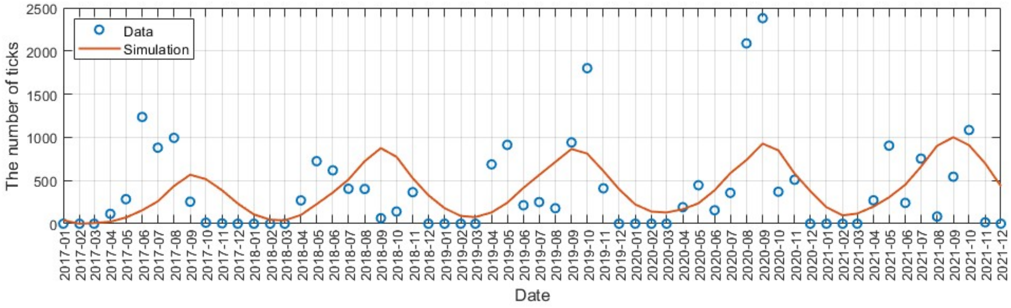


**Fig A: Parameter estimation results for climate-dependent parameter** Collected actual data (blue circle) and estimation result (red line) of the abundance of the total tick population including larvae, nymphs, and adults.

## 2 Estimation of control parameter, $CR_1, CR_2$

Similar to the previous section, to estimate each control parameter $CR_1(t)$ and $CR_2(t)$, we used *lsqcurvefit* in MATLAB to fit an exponential function to the data obtained through experiments at Jang *et al.* [1] and Lee *et al.* [2], respectively. For each control parameter, the fitted exponential functions are $CR_1(t) = 0.91 \times e^{-0.13t}$ and $CR_2(t) = 2.53 \times e^{-0.79t}$.

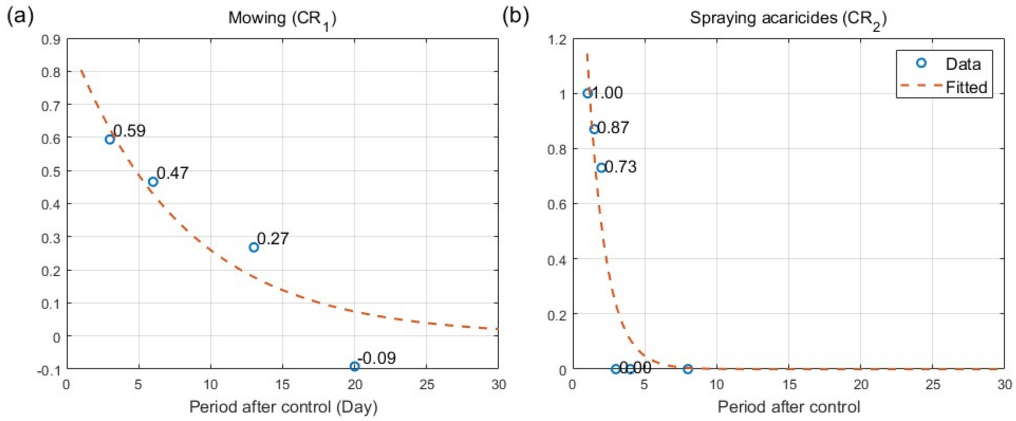


**Fig B: The fitting results of control parameters** (a) $CR_1(t)$ and (b) $CR_2(t)$

# 3 Estimation of Cost

To adjust the biting rate according to temperature, we defined it in the main text as $C_\eta$. As we mentioned in the main text, most of the ticks finished their questing behavior at $15°C$, and this behavior continues up to $35°C$ [3, 4]. Therefore, we fit a temperature-dependent parameter, $C_\eta$, to a sigmoid function, $C_\eta(T) = \frac{0.99}{1+e^{-0.82\times(T-9)}}$ that converges to 1 after passing a certain threshold. The fitting result of $C_\eta$ is in Fig C (a). In addition, since the medical cost and wage loss differ by age group, each patient was assigned an age group using a gamma distribution that reflects the actual patient age distribution in 2022, $Gamma(4.51, 0.39)$. The actual patient age distribution and fitted probability density function are in Fig C (b). We simulated 100 times to assign the patients to age groups and calculate the medical cost and wage loss in each simulation. After 100 times simulations, the respective averages of medical costs and wage losses become the final medical cost and wage loss for each control measure scenario.

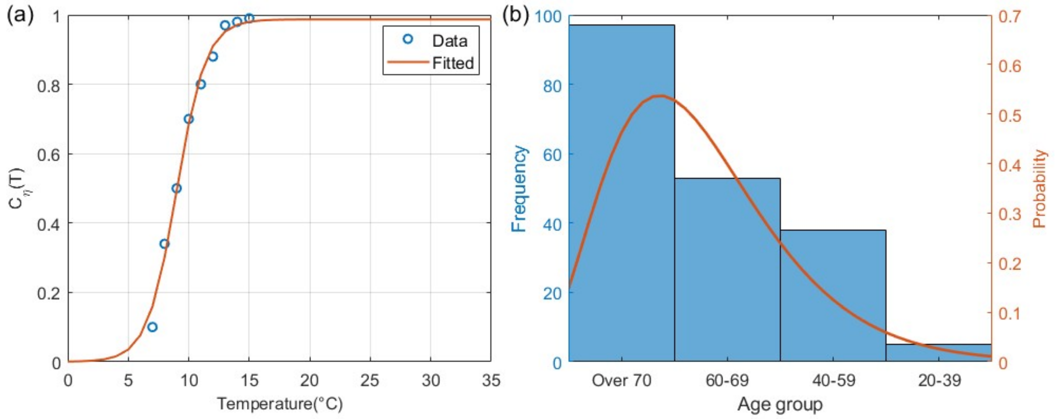


**Fig C: The parameter fitting results for cost estimation** (a) The fitted results of $C_\eta(T)$ [3,4] (b) The age distribution of SFTS patients and fitted probability density function [5].

# References


[1] Jang CW, Kim H, Kim GH, Lee HI. Evaluation of effectiveness of mowing works for integrated tick control method. Public Health Weekly Report. 2021;14(44):3103–3110.

[2] Lee DW, Chang KS, Kim MJ, Ahn YJ, Jo HC, Kim SI. Acaricidal activity of commercialized insecticides against *Haemaphysalis longicornis* (Acari: *Ixodidae*) nymphs. Journal of Asia-Pacific Entomology. 2015;18(4):715–718.

[3] Gilbert L, Aungier J, Tomkins JL. Climate of origin affects tick (*Ixodes ricinus*) host-seeking behavior in response to temperature: implications for resilience to climate change? Ecology and evolution. 2014;4(7):1186–1198.

[4] Nielebeck C, Kim SH, Pepe A, Himes L, Miller Z, Zummo S, et al. Climatic stress decreases tick survival but increases rate of host-seeking behavior. Ecosphere. 2023;14(1):e4369.

[5] Choi JH, Hwang JH, Lee HS, Hwang KW. Epidemiological Characteristics of Cases and Deaths of Severe Fever with Thrombocytopenia Syndrome (SFTS), 2022. Public Health Weekly Report. 2023;16(30):1025–1037.
