# Supplementary material for: The impact of climate change on ecology of tick associated with tick-borne diseases
Source: PLoS Comput Biol. 2025 Apr 8;21(4):e1012903. doi: 10.1371/journal.pcbi.1012903 (PMC12002643; doi:10.1371/journal.pcbi.1012903)
Supplement: S1 Table — (PDF) [file pcbi.1012903.s002.pdf]

S1 Table: The formula of development rates and its coefficients.

The development rate	Formula	Coefficient
$d_1(T, H)$	$\max\{0, a_0T^2 + a_1H^2 + a_2T \cdot H + a_3T + a_4H + a_5\}$	$[a_0, a_1, a_2, a_3, a_4, a_5]$ = [2.1674e-14, 1.2551e-15, 0.00014035, 3.1808e-13, 6.3242e-08, 1.6433e-05]
$d_2(T)$	$\max\{0, a_6T^2 + a_7T + a_8\}$	$[a_6, a_7, a_8]$ = [0.00040815, 0.0012284, -0.10391]
$d_3(T)$	$\max\{0, a_9T^2 + a_{10}T + a_{11}\}$	$[a_9, a_{10}, a_{11}]$ = [2.4696e-10, 1.0003e-08, 0.17439]
$d_4(T)$	$\max\{0, a_{12}T^2 + a_{13}T + a_{14}\}$	$[a_{12}, a_{13}, a_{14}]$ = [6.2836e-17, 3.1928e-15, 5.8214e-05]
