# Supplementary material for: The impact of climate change on ecology of tick associated with tick-borne diseases
Source: PLoS Comput Biol. 2025 Apr 8;21(4):e1012903. doi: 10.1371/journal.pcbi.1012903 (PMC12002643; doi:10.1371/journal.pcbi.1012903)
Supplement: S2 Text — (PDF) [file pcbi.1012903.s003.pdf]

S2 Text. Initial condition of simulation

Temperature projections of the SSP scenario were started in 2021. To set the initial conditions, we first compared the 2021 data, which is an overlapping period between the data fitting period and the SSP temperature forecast period. As we can see in Fig A, the projected temperatures are higher than the real temperature for most months in 2021. Therefore, we assumed that the observed climate in 2021 may not match the actual change in the early stage of climate change, and assumed that the observed climate in 2021 will continue until 2030. In addition, in order to check the change in tick population after the mid-stage of climate change, we assumed 2030, which is arbitrarily close to the future but can be considered mid-stage, as the simulation start date. As explained above, the simulation was conducted from 2021 to 2029 based on the 2021 climate data. After that, the population of each tick stage among the simulation results in December 2029 was set as the initial condition for the simulation from 2030 to 2100.

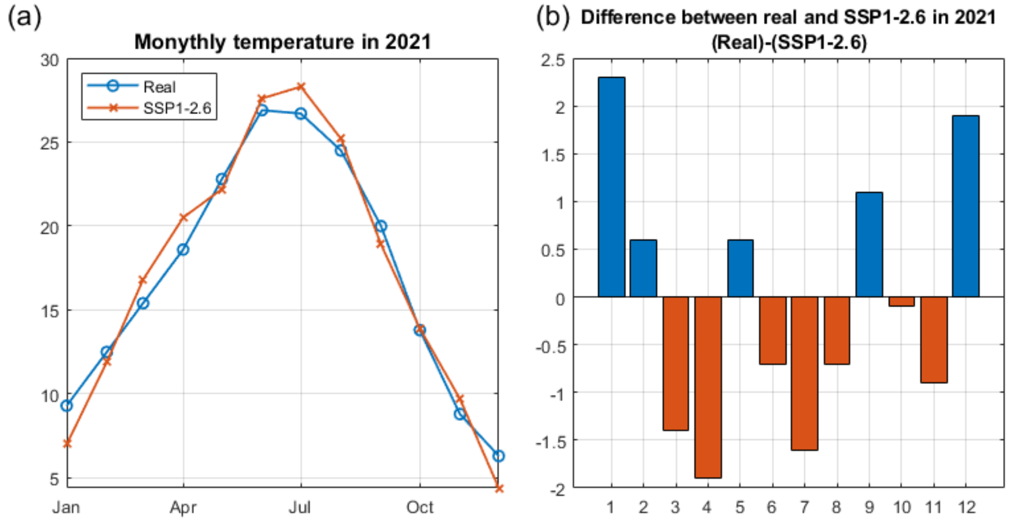

Fig A: Comparison of the real and SSP1-2.6 average temperatures in 2021. (a) Compare of real and projection of SSP1-2.6 monthly temperature in 2021 (b) Difference between real and SSP1-2.6 in 2021

Comparing the temperature in 2021 with the actual measured temperature in 2022 and 2023, we found that the temperature in 2021 was higher for most months. Since the 2021 temperature will be maintained between 2021 to 2030, the tick population will gradually increase until 2030. In addition, if we compare the SSP1-2.6 temperature data from 2030 to 2032 with the 2021 temperature data, we can see that 2021 temperature data is much higher (At most almost 4℃) in most months except for July and August, which are mid-summer (See the Fig B). Therefore, if we start simulation using the SSP1-2.6 temperature data from 2030, the tick population shows a decreasing trend in the already increased number of ticks.

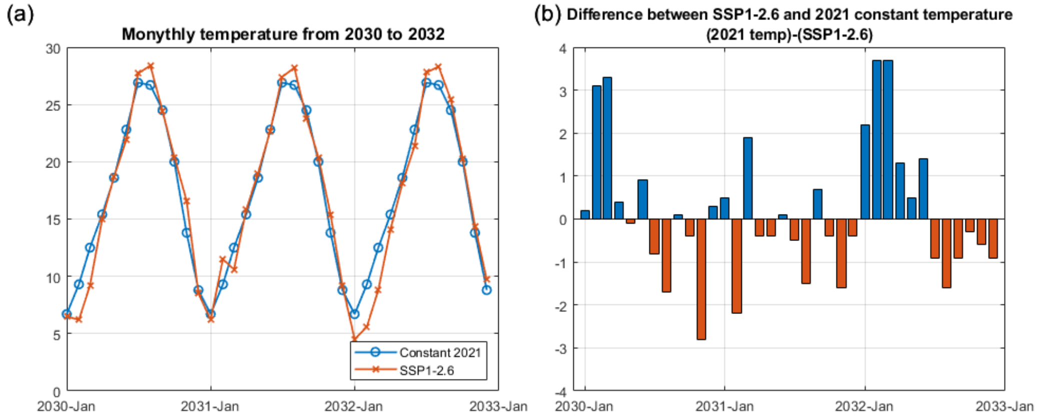

Fig B: Comparison of the real(2021) and SSP1-2.6 average temperatures from 2030 to 2032. (a) Compare the 2021 temperature and projection of SSP1-2.6 monthly temperature from 2030 to 2032 (b) Difference between 2021 temperature and SSP1-2.6 from 2030 to 2032
