# Supplementary material for: The impact of climate change on ecology of tick associated with tick-borne diseases
Source: PLoS Comput Biol. 2025 Apr 8;21(4):e1012903. doi: 10.1371/journal.pcbi.1012903 (PMC12002643; doi:10.1371/journal.pcbi.1012903)
Supplement: S2 Table — (PDF) [file pcbi.1012903.s004.pdf]

S2 Table: **Cumulative number of the collected ticks in 2100 when each control measure is implemented 1M and 2M.** For the 2M scenario, each control measure is implemented for two consecutive months from the starting month to the following 1 month. For example, for 2M, if the control measure is implemented starting in April, the control measure will be implemented in April and May. For each control measure, the results in bold indicate the minimum cumulative abundance of ticks, or in other words, the results of the most effective control measure.

			Starting month implementation of control measure							
			Apr	May	June	July	Aug	Sep	Oct	Nov
1M	SSP1-2.6	No control measure	13872452							
		Mowing	91608	85989	80310	**79999**	85803	93100	98764	99849
		Spraying acaricide	67159	**65993**	67640	72545	77800	80693	80531	78867
		Both	61559	57639	**55875**	59362	67075	74325	77972	77096
	SSP2-4.5	No control measure	17644313							
		Mowing	114788	106741	98770	**98665**	106934	117641	125700	127526
		Spraying acaricide	79834	**78425**	80944	87994	95413	99574	99321	96876
		Both	71873	66801	**64776**	69774	80296	90527	95506	94324
	SSP3-7.0	No control measure	24706068							
		Mowing	156362	144081	**132139**	132460	145296	162020	175519	178739
		Spraying acaricide	100768	**99308**	103717	114721	126318	132736	132357	128254
		Both	88174	81585	**79324**	87105	102775	118143	126039	124067
	SSP5-8.5	No control measure	30726247							
		Mowing	194024	177790	161789	**161530**	177799	200106	218687	224244
		Spraying acaricide	119003	**117046**	122671	136988	152503	161587	161678	156100
		Both	102484	94103	**91056**	100613	120493	140925	152234	150201
2M	SSP1-2.6	No control measure	13872452							-
		Mowing	77384	68387	**63928**	67854	78275	89710	96081	
		Spraying acaricide	**48622**	48782	52497	58867	64493	66575	65456	
		Both	40929	**37836**	38333	43944	52833	60198	62493	
	SSP2-4.5	No control measure	17644313							-
		Mowing	93911	81590	**75827**	81478	96161	112412	121690	
		Spraying acaricide	**54918**	55348	60381	68976	76691	79535	77884	
		Both	44926	**41342**	42262	49472	61038	70824	73733	
	SSP3-7.0	No control measure	24706068							-
		Mowing	122436	104445	**96526**	105394	127565	153108	168567	
		Spraying acaricide	**64019**	65192	72733	85351	96695	100907	98248	
		Both	50126	**46098**	47755	57579	73498	87566	91854	
	SSP5-8.5	No control measure	30726247							-
		Mowing	148426	124861	**113993**	124534	152827	186809	208866	
		Spraying acaricide	**71280**	72641	81870	97831	112886	119104	115947	
		Both	54332	**49412**	51080	62532	82106	100431	106840	
