# Supplementary material for: The impact of climate change on ecology of tick associated with tick-borne diseases
Source: PLoS Comput Biol. 2025 Apr 8;21(4):e1012903. doi: 10.1371/journal.pcbi.1012903 (PMC12002643; doi:10.1371/journal.pcbi.1012903)
Supplement: S3 Table — (PDF) [file pcbi.1012903.s005.pdf]

S3 Table: Cumulative abundance of ticks in 2100 when each control measure is implemented 3M and 4M. For the 3M(4M) scenario, each control measure is implemented for three(four) consecutive months from the starting month to the following 2(3) months. For example, for 3M, if the control measure is implemented starting in April, the control measure will be implemented in April, May, and July. For each control measure, the results in bold indicate the minimum cumulative abundance of ticks, or in other words, the results of the most effective control measure.

			Starting month implementation of control measure							
			Apr	May	June	July	Aug	Sep	Oct	Nov
3M	SSP1-2.6	No control measure	13872452						-	
		Mowing	62162	55199	**54922**	62351	75551	87278		
		Spraying acaricide	**38564**	40312	44850	50914	55293	56257		
		Both	29543	**28402**	30698	36911	45038	50566		
	SSP2-4.5	No control measure	17644313						-	
		Mowing	72674	63744	**63698**	73962	92108	108829		
		Spraying acaricide	**42277**	44600	50488	58373	64170	65330		
		Both	31411	**30353**	33211	40743	50886	57943		
	SSP3-7.0	No control measure	24706068						-	
		Mowing	90091	**77950**	78362	93613	120982	147171		
		Spraying acaricide	**47029**	50450	58536	69509	77620	79177		
		Both	33429	**32533**	36208	45700	58951	68475		
	SSP5-8.5	No control measure	30726247						-	
		Mowing	105804	89892	**89715**	108268	143212	178505		
		Spraying acaricide	**50230**	54097	63778	77457	88179	90608		
		Both	34596	**33373**	37285	47988	63874	76171		
4M	SSP1-2.6	No control measure	13872452					-		
		Mowing	50637	**47865**	50787	60312	73584			
		Spraying acaricide	**33087**	35677	40152	45116	48189			
		Both	**23358**	23836	26903	32781	39385			
	SSP2-4.5	No control measure	17644313					-		
		Mowing	57473	**54187**	58315	71177	89363			
		Spraying acaricide	**35809**	39019	44557	50798	54709			
		Both	**24475**	25165	28879	35797	43708			
	SSP3-7.0	No control measure	24706068					-		
		Mowing	68291	**64284**	70438	89245	116573			
		Spraying acaricide	**38767**	43060	50341	58669	63825			
		Both	**25537**	26562	30961	39268	49151			
	SSP5-8.5	No control measure	30726247					-		
		Mowing	77473	**71996**	79065	102137	137228			
		Spraying acaricide	**40402**	45155	53706	63905	70618			
		Both	**25871**	26795	31291	40470	52062			
