# Supplementary material for: The impact of climate change on ecology of tick associated with tick-borne diseases
Source: PLoS Comput Biol. 2025 Apr 8;21(4):e1012903. doi: 10.1371/journal.pcbi.1012903 (PMC12002643; doi:10.1371/journal.pcbi.1012903)
Supplement: S1 Fig — (PDF) [file pcbi.1012903.s006.pdf]

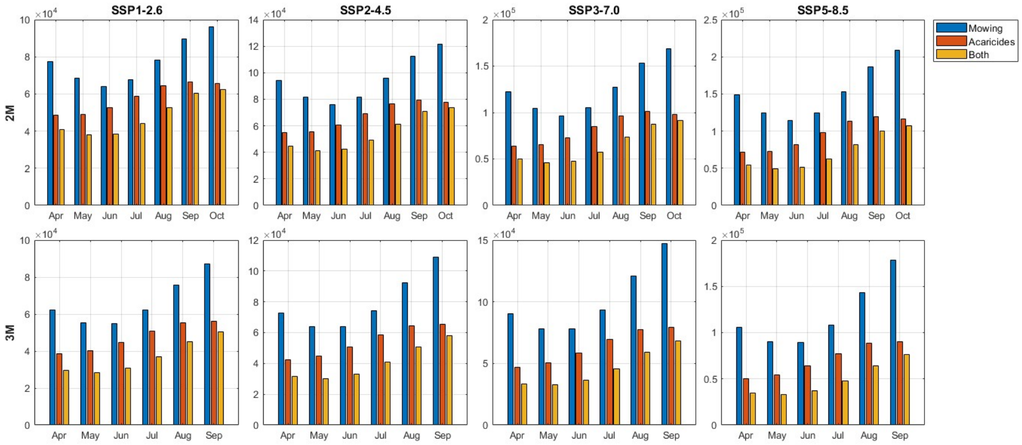

S1 Fig: **Cumulative number of the collected ticks when each control measure is implemented for 2M and 3M.** For the 2M(3M) scenario, each control measure is implemented for two(three) consecutive months from the starting month to the following 1(2) months. For example, for 2M, if the control measure is implemented starting in April, the control measure will be implemented in April, May.
