# Supplementary material for: The impact of climate change on ecology of tick associated with tick-borne diseases
Source: PLoS Comput Biol. 2025 Apr 8;21(4):e1012903. doi: 10.1371/journal.pcbi.1012903 (PMC12002643; doi:10.1371/journal.pcbi.1012903)
Supplement: S2 Fig — (PDF) [file pcbi.1012903.s007.pdf]

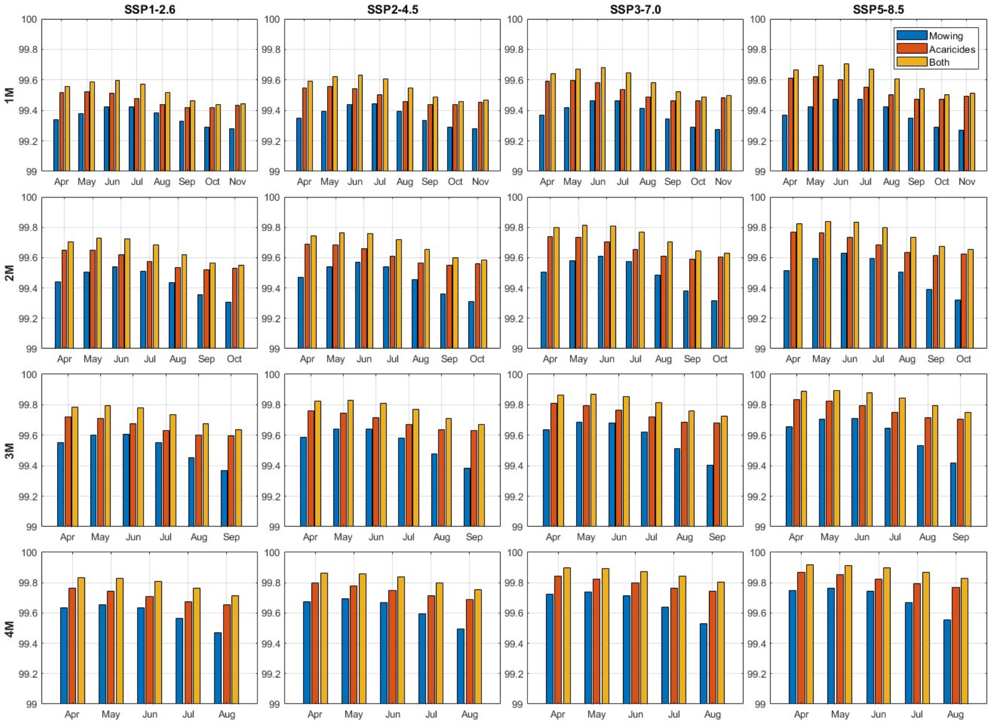

S2 Fig: **Reduction rate of each control measure for different SSP scenarios.** Each bar graph means the reduction rate divided by the difference between the cumulative tick abundance with each control measure and no control by the cumulative tick abundance with no control, then multiplied by 100.
