# Supplementary material for: The impact of climate change on ecology of tick associated with tick-borne diseases
Source: PLoS Comput Biol. 2025 Apr 8;21(4):e1012903. doi: 10.1371/journal.pcbi.1012903 (PMC12002643; doi:10.1371/journal.pcbi.1012903)
Supplement: S3 Fig — (PDF) [file pcbi.1012903.s008.pdf]

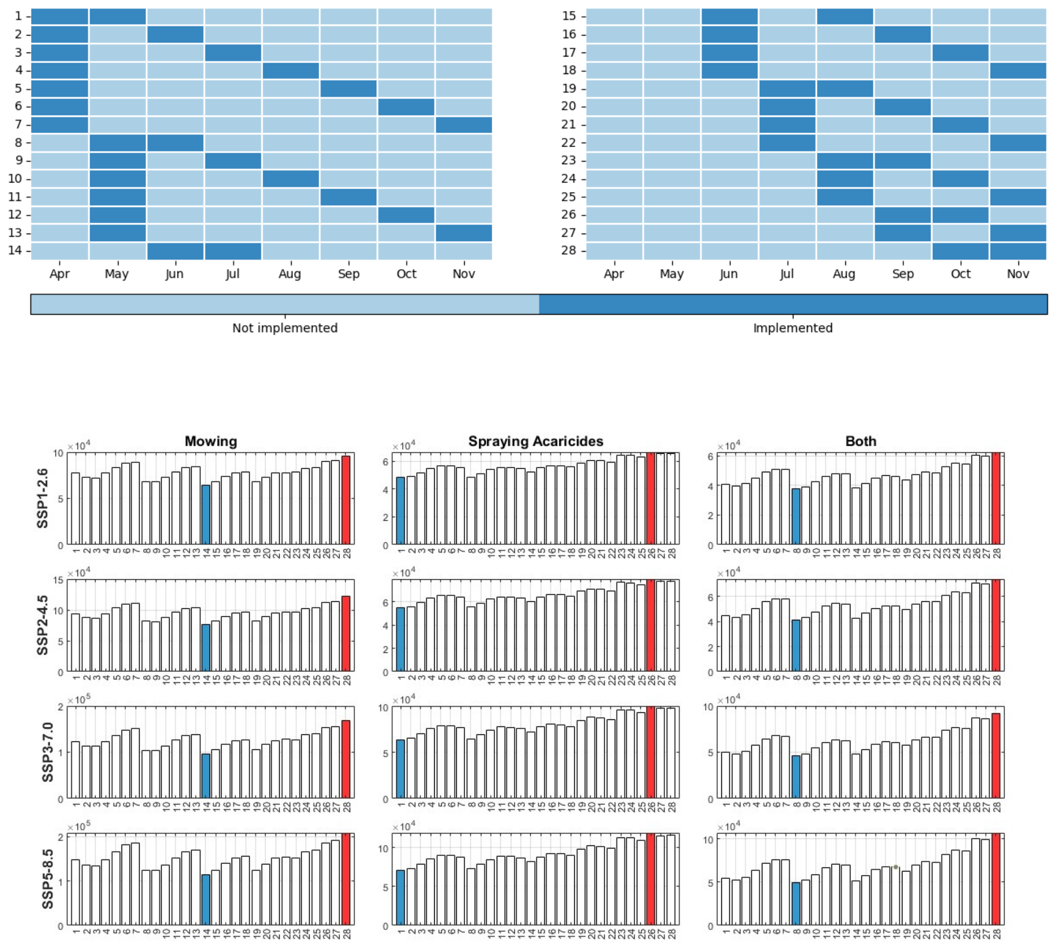

S3 Fig: **Simulation results including all possible control measure combinations based on the 2M scenario.** Top: Scenarios when each control measure is implemented twice a year (2M). The y-axis denotes the scenario number. Bottom: Cumulative abundance of ticks when each control measure is implemented twice a year (2M). The x-axis denotes the scenario number. The blue bar is the minimum cumulative abundance, which means the most effective control measure scenario. The red bar is the maximum cumulative abundance, which means the least effective control measure scenario.
