# Supplementary material for: The impact of climate change on ecology of tick associated with tick-borne diseases
Source: PLoS Comput Biol. 2025 Apr 8;21(4):e1012903. doi: 10.1371/journal.pcbi.1012903 (PMC12002643; doi:10.1371/journal.pcbi.1012903)
Supplement: S4 Table — (PDF) [file pcbi.1012903.s011.pdf]

S4 Table: **Starting month in which each control measure is implemented when simulating the Best and Worst scenarios according to SSP scenarios.** For the 2M(3M,4M) scenario, each control measure is implemented for two(three, four) consecutive months from the starting month to the following 1(2,3) months. For example, for 2M, if the control measure is implemented starting in April, the control measure will be implemented in April and May.

		Starting month implementation of control measure					
		Mowing		Spraying acaricide		Both	
		Best	Worst	Best	Worst	Best	Worst
SSP1-2.6	1M	July	Nov	May	Sep	June	Oct
	2M	June	Oct	Apr	Sep	May	Oct
	3M	June	Sep	Apr	Sep	May	Sep
	4M	May	Aug	Apr	Aug	Apr	Aug
SSP2-4.5	1M	July	Nov	May	Sep	June	Oct
	2M	June	Oct	Apr	Sep	May	Oct
	3M	June	Sep	Apr	Sep	May	Sep
	4M	May	Aug	Apr	Aug	Apr	Aug
SSP3-7.0	1M	June	Nov	May	Sep	June	Oct
	2M	June	Oct	Apr	Sep	May	Oct
	3M	May	Sep	Apr	Sep	May	Sep
	4M	May	Aug	Apr	Aug	Apr	Aug
SSP5-8.5	1M	July	Nov	May	Oct	June	Oct
	2M	June	Oct	Apr	Sep	May	Oct
	3M	June	Sep	Apr	Sep	May	Sep
	4M	May	Aug	Apr	Aug	Apr	Aug
