# Supplementary material for: The impact of climate change on ecology of tick associated with tick-borne diseases
Source: PLoS Comput Biol. 2025 Apr 8;21(4):e1012903. doi: 10.1371/journal.pcbi.1012903 (PMC12002643; doi:10.1371/journal.pcbi.1012903)
Supplement: S6 Fig — (PDF) [file pcbi.1012903.s012.pdf]

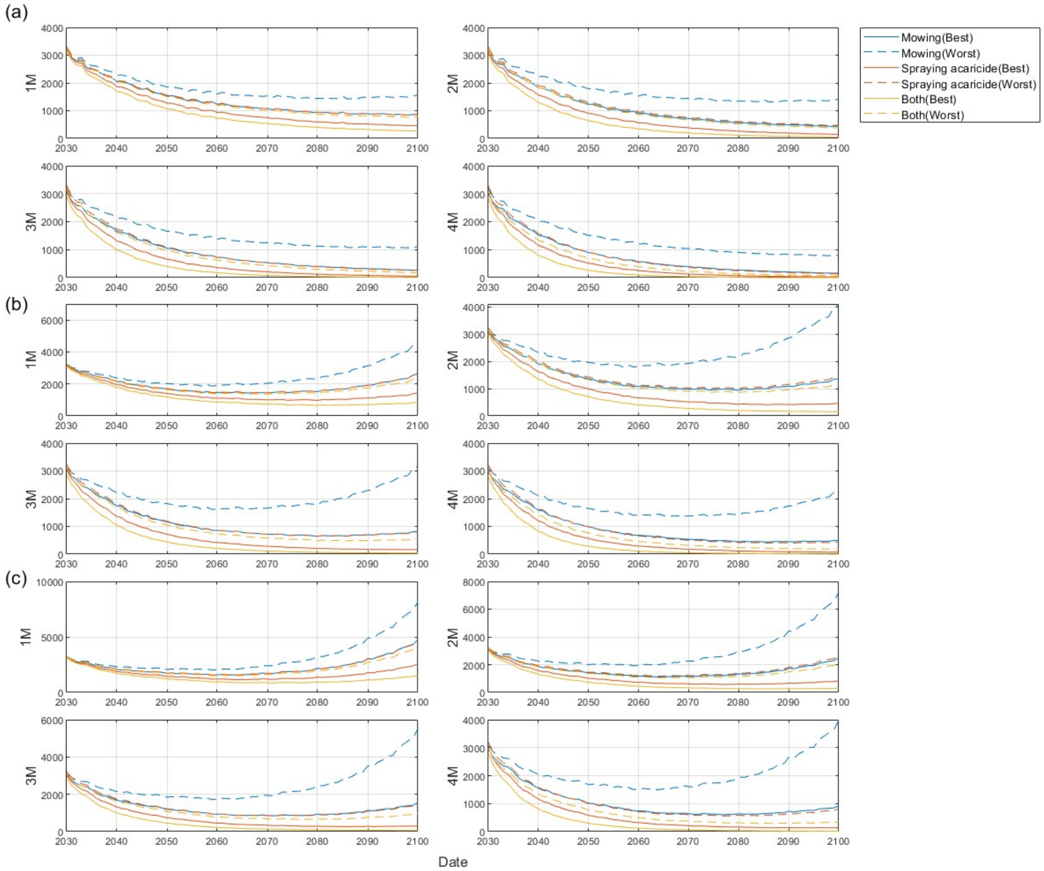

S6 Fig: **The annual tick abundance according to the best and worst scenarios for each control measure.** The annual tick abundance according to the best and worst scenarios for each control measure under the (a) SSP1-2.6 (b) SSP2-4.5 (c) SSP3-7.0. Best: Control measure scenario that reduces the tick population the most. Worst: Control measure scenario that reduces the tick population the least.
