# Supplementary material for: The impact of climate change on ecology of tick associated with tick-borne diseases
Source: PLoS Comput Biol. 2025 Apr 8;21(4):e1012903. doi: 10.1371/journal.pcbi.1012903 (PMC12002643; doi:10.1371/journal.pcbi.1012903)
Supplement: S9 Fig — (PDF) [file pcbi.1012903.s015.pdf]

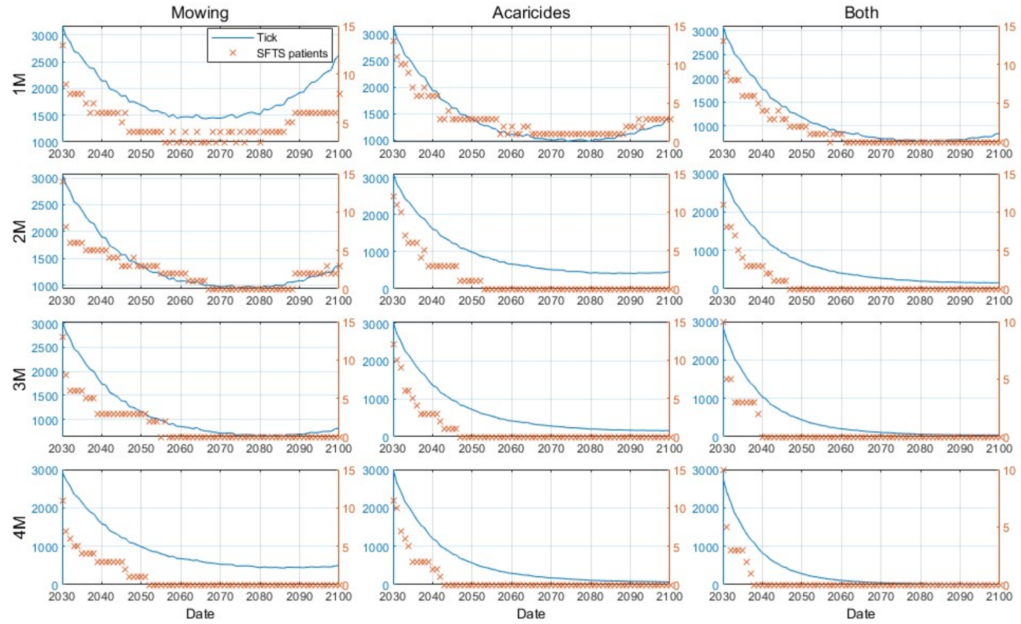

S9 Fig: **The annual tick abundance and SFTS incidence under SSP3-7.0 scenario.** The annual tick abundance and SFTS incidence according to control measure and the duration of control measure implementation per year under SSP3-7.0 scenario.
