# Supplementary material for: The impact of climate change on ecology of tick associated with tick-borne diseases
Source: PLoS Comput Biol. 2025 Apr 8;21(4):e1012903. doi: 10.1371/journal.pcbi.1012903 (PMC12002643; doi:10.1371/journal.pcbi.1012903)
Supplement: S10 Fig — (PDF) [file pcbi.1012903.s016.pdf]

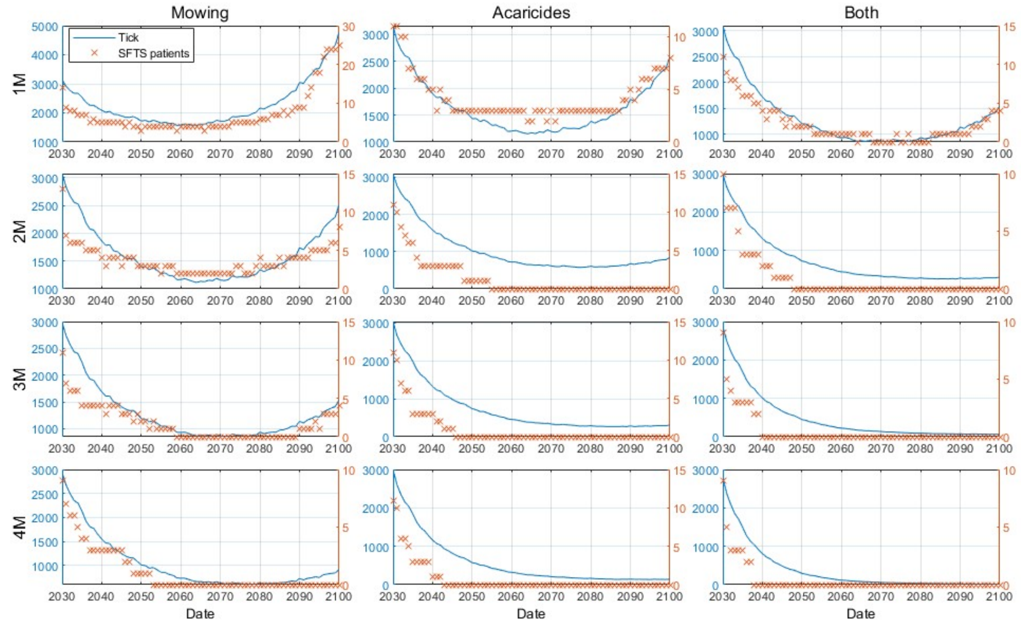

S10 Fig: **The annual tick abundance and SFTS incidence under SSP5-8.5 scenario.** The annual tick abundance and SFTS incidence according to control measure and the duration of control measure implementation per year under SSP5-8.5 scenario.
