# Supplementary material for: The impact of climate change on ecology of tick associated with tick-borne diseases
Source: PLoS Comput Biol. 2025 Apr 8;21(4):e1012903. doi: 10.1371/journal.pcbi.1012903 (PMC12002643; doi:10.1371/journal.pcbi.1012903)
Supplement: S11 Fig — (PDF) [file pcbi.1012903.s017.pdf]

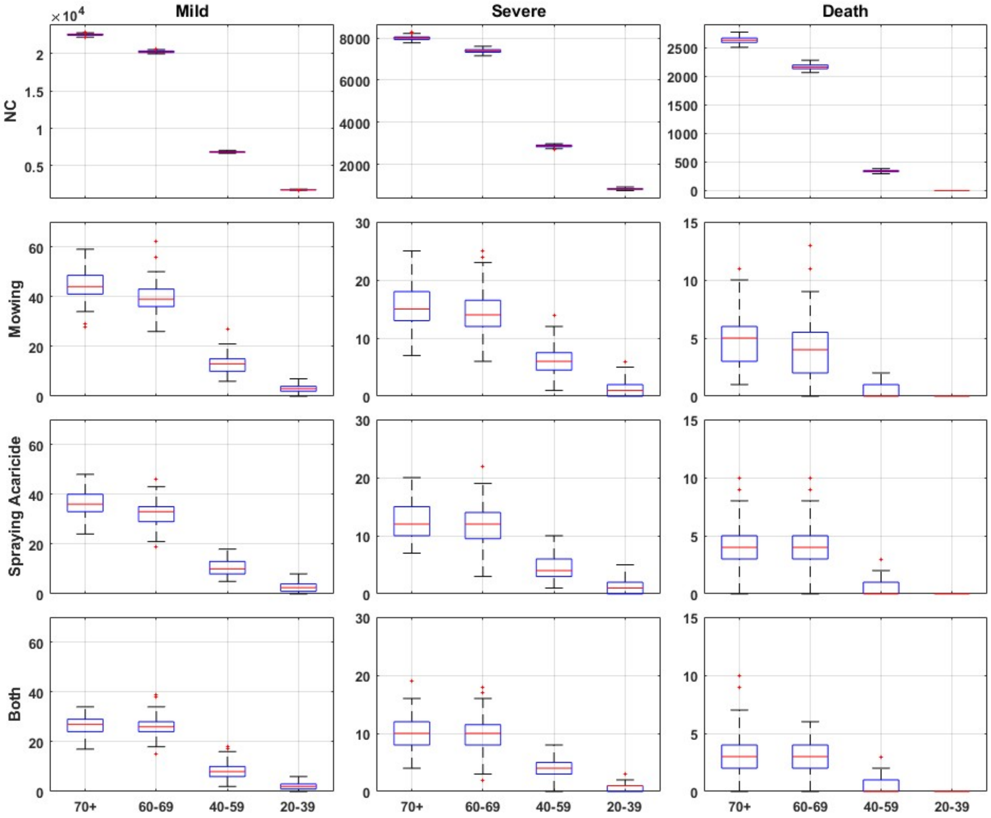

S11 Fig: **The plot of SFTS patients by severity under SSP1-2.6 scenario**. For the control measures scenario, each control measure is implemented once per year.
