# Supplementary material for: The impact of climate change on ecology of tick associated with tick-borne diseases
Source: PLoS Comput Biol. 2025 Apr 8;21(4):e1012903. doi: 10.1371/journal.pcbi.1012903 (PMC12002643; doi:10.1371/journal.pcbi.1012903)
Supplement: S3 Text — (PDF) [file pcbi.1012903.s021.pdf]

S3 Text: Cost analysis

We conducted a Cost-Benefit analysis of the total cost and the medical cost. The cost-benefit index for total cost is calculated by the following formula [1]:

$$I_1^{C_{n,m}} = \frac{T_N - T_{C_{n,m}}}{C_{C_{n,m}}},$$

where T_N is the total cost for no control measure, $T_{C_{n,m}}$ is the total cost for when the control measures type n implemented for m months, and $C_{C_{n,m}}$ is the control measures cost for when the control measures type n implemented for m months.
Similarly, the cost-benefit index for medical cost is calculated by the following formula [1]:

$$I_2^{C_{n,m}} = \frac{M_N - M_{C_{n,m}}}{C_{C_{n,m}}},$$

where M_N is the medical cost for no control measure, $M_{C_{n,m}}$ is the medical cost for when the control measures type n implemented for m months, and $C_{C_{n,m}}$ is the control measures cost for when the control measures type n implemented for m months.

Fig A and Fig B show the cost-benefit index for total($I_1^{C_{n,m}}$) and medical costs($I_2^{C_{n,m}}$) associated with control measures and climate change scenarios, respectively. According to Fig A and Fig B, as the duration of the control measure is longer, the benefit of costs decreases compared to the cost of control measures. However, it is obvious that implementing control measures, although for the 4M scenario, is still cost-beneficial compared to not implementing any control measures for both total cost and medical cost.

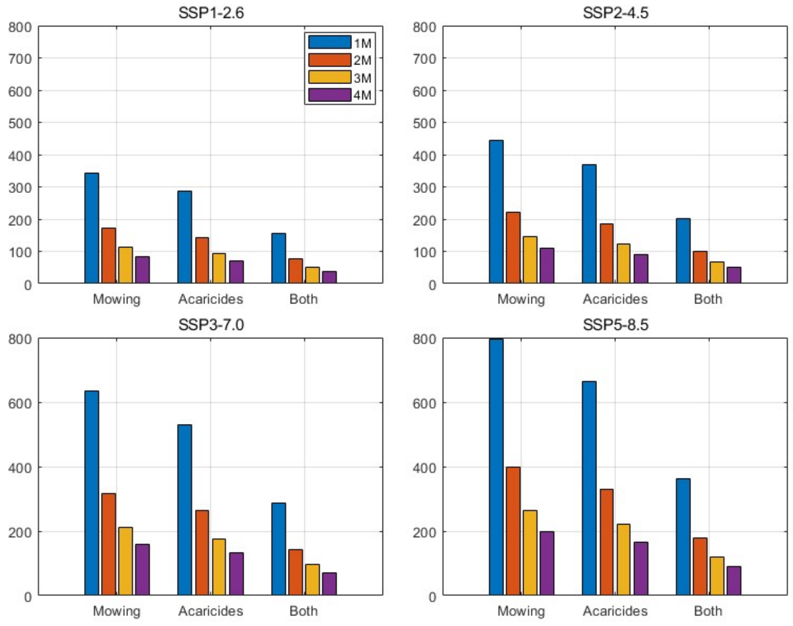

Fig A: Cost-Benefit analysis for total cost Cost-Benefit analysis for total cost($I_1^{C_{n,m}}$) for each control measure and climate change scenario.

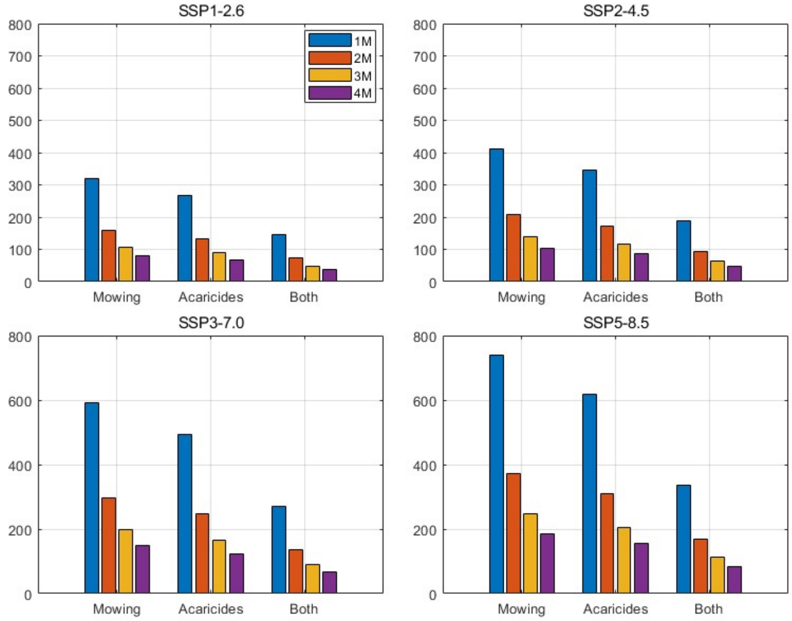

Fig B: Cost-Benefit analysis for medical cost Cost-Benefit analysis for medical cost($I_2^{C_{n,m}}$) for each control measure and climate change scenario.

Furthermore, we introduce a composite measure that quantifies the reduction index in SFTS cases per unit cost of control implementation:

$$I_3^{C_{n,m}} = \frac{N_N^{SFTS} - N_{C_{n,m}}^{SFTS}}{C_{C_{n,m}}},$$

where N_N^{SFTS} is the number of SFTS in the absence of any control measures, $N_{C_{n,m}}^{SFTS}$ is the number of SFTS patients when the control measure type n is implemented for m months, and $C_{C_{n,m}}$ is the cost associated with implementing control measure type n for m months. Fig C illustrates the reduction index in SFTS cases per unit cost of control implementation ($I_3^{C_{n,m}}$) associated with control measures and climate change scenarios. Like the results of $I_1^{C_{n,m}}$ and $I_2^{C_{n,m}}$, the reduction index of SFTS patients by control measure cost has a positive value regardless of the type and duration of the control measure. Furthermore, our results indicate that the efficacy of control measures increases under scenarios of worsening climate change.

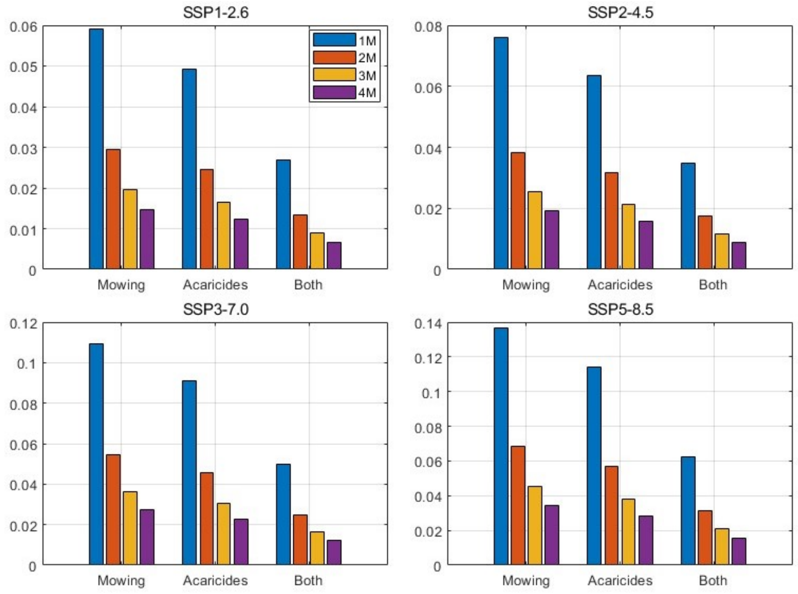

Fig C: The reduction rate of SFTS patients by control measure cost The reduction rate of SFTS patients by control measure cost($I_3^{C_{n,m}}$) for each control measure and climate change scenario.

References

[1] Ramos DG, Arezes PM, Afonso P. Analysis of the return on preventive measures in musculoskeletal disorders through the benefit–cost ratio: A case study in a hospital. International Journal of Industrial Ergonomics. 2017;60:14–25.
