# Supplementary material for: The impact of climate change on ecology of tick associated with tick-borne diseases
Source: PLoS Comput Biol. 2025 Apr 8;21(4):e1012903. doi: 10.1371/journal.pcbi.1012903 (PMC12002643; doi:10.1371/journal.pcbi.1012903)
Supplement: S4 Fig — (PDF) [file pcbi.1012903.s009.pdf]

List of answers to the reviewer 1's comments

We are grateful for the valuable comments from the reviewer and the opportunity to generate a revised manuscript. In this revision, we addressed all of the reviewer's comments and have made an effort to better explain the relevance of our results.

Overall Comment

Overall I really like this paper, it does some interesting investigations and most comments are small editorial changes for clarity. However, there are three things which are more fundamental and might mean issues with the model. These need addressing before publication, either by more justification in the text or they potentially require correction if I have understood what is happening correctly.

Point by point response

Main comments

1 Page 4 line 128 you say you calculate K based on hosts per m2 for grass and wooded areas and then add them, but I don't think that is what you should do. Surely you should do the average, or run the model on the two types of area, but adding them gives you a much bigger K than you would expect and will impact on all of your results going forward.

(Answer) Thank you for the comment. The patch model, considering distinct wood and grass areas, was our initial approach. However, precise location data for tick collection are unavailable. Thus, to estimate carrying capacity, we opted to utilize the total grass and wood area on Jeju Island. The formula employed for this calculation is as follows:

$$\boldsymbol{K = M \times (A_W \times C_w + A_G \times C_G)}$$

This calculated carrying capacity (K) has been incorporated into Eq. (1) and Table 1 of the manuscript. While acknowledging the limitations of using total island area due to the lack of precise collection points, we believe this approach provides a reasonable estimate of K for the model, given the available data.

Parameter	Definition	Value	Reference
M	Maximum number of ticks per host	200	[1]
A_W	Woods area in Jeju Island (m^2)	847180000	[2]
C_w	Carrying capacity for host per m^2 in wood area	0.002	[1]
A_G	Grass area in Jeju Island (m^2)	154550000	[3]
C_G	Carrying capacity for host per m^2 in grass area	0.001	[1]

Table R1 Parameter description for the carrying capacity ***(this part has been newly added in Table 1 of the main manuscript.)***

[Reference]

1. Gaff, Holly D., and Louis J. Gross. "Modeling tick-borne disease: a metapopulation model." ***Bulletin of Mathematical Biology*** **69 (2007): 265-288.**

2. Jeju City Civil Service Office. Forests and fields area generalization (unit: Ha); 2022. https://www.jejusi.go.kr/information/open/data/view.do?id=447.

3. Kim S. There is an urgent need for measures to reduce grassland in Jeju every year due to various development projects.; 2023. https://www.jejunews.com/news/articleView.html?idxno=2199710.

2 Page 5- the model: The functional forms of the development rates in your model come from fit to data I think, but did you consider making them mechanistic? For example, you know that for eggs higher humidity leads to higher hatchability rate and lower development time so you could include that specifically but that does not seem to be D1. I don't think what you have done is wrong but you should justify that choice when you have specific data that would allow you to do something mechanistic.

(Answer) Thank you for the comment. While our current model employs functional forms derived from fitting empirical data, we considered incorporating mechanistic relationships where possible: Firstly, while we considered incorporating mechanistic relationships based on cited literature, as

described in lines 81-90, our current model prioritizes accurately capturing the observed overall trends in development rates across various temperature and humidity conditions. Our fitting procedure, as shown in Figure 3(b), successfully reproduces these trends, where all development and reproduction rates increase with rising temperature. This is consistent with findings from previous studies. Secondly, as correctly pointed out, the development rate from eggs to larvae, $d_1(T, H)$, indeed exhibits a positive relationship with relative humidity as well. Furthermore, the coefficients related to temperature and relative humidity in $d_1(T, H)$ as presented in S1 Table, are all positive, aligning qualitatively with established research. This suggests that our model, although derived from data fitting, implicitly captures the general influence of humidity on this specific development stage.

However, explicitly incorporating a mechanistic relationship for humidity in $d_1(T, H)$ would necessitate a level of detail and complexity that is not currently supported by the available data. While we know higher humidity generally leads to higher hatchability and faster development, the precise quantitative relationship, particularly in interaction with temperature, is not well established for this specific species and would require targeted experiments.

Our current dataset, while sufficient to establish the observed correlations and inform the phenomenological model, lacks the granularity necessary to confidently parameterize a fully mechanistic model. Specifically, data limitations preclude us from definitively determining the precise functional relationship between humidity and $d_1(T, H)$ beyond the observed positive correlation. Future research, incorporating targeted experiments designed to elucidate these specific mechanistic relationships, would be invaluable for refining the model and enhancing its predictive power. For the present study, given the data constraints, the chosen approach represents a balance between capturing the essential dynamics and avoiding over-parameterization. We believe this approach provides a robust and useful framework for understanding the overall impact of climate on the organism's life cycle, while acknowledging the limitations and potential for future mechanistic development.

3 Parameter estimation, this bit seems key. You say you fit it using the data in figure 1c but I think that means each development rate is fitted to a different set of data ie larvae, nymphs and adults? So you could give a bit more detail here and explain that.

(Answer) Thank you for the comment. The reviewer correctly points out that our statement about fitting the model using data in Figure 1c might be interpreted as implying that each developmental rate (larva, nymph, and adult) was fitted independently to separate datasets.

To clarify, we have provided Figure R1 below, which illustrates the fitting results specifically for the larval stage. This stage is emphasized because larvae constituted the overwhelmingly dominant proportion of the collected tick population.

However, we acknowledge the inherent difficulty in achieving precise, stage-specific data fitting, given the constraints of limited data and the reliance on numerical approximation methods. This limitation was recognized in the main manuscript in lines 456-459, as stated:

"Even within the limited data available, the number of collected larvae is dominantly greater than the number of ticks in other stages, it is difficult to estimate the exact number of ticks in each stage by numerical approximation methods."

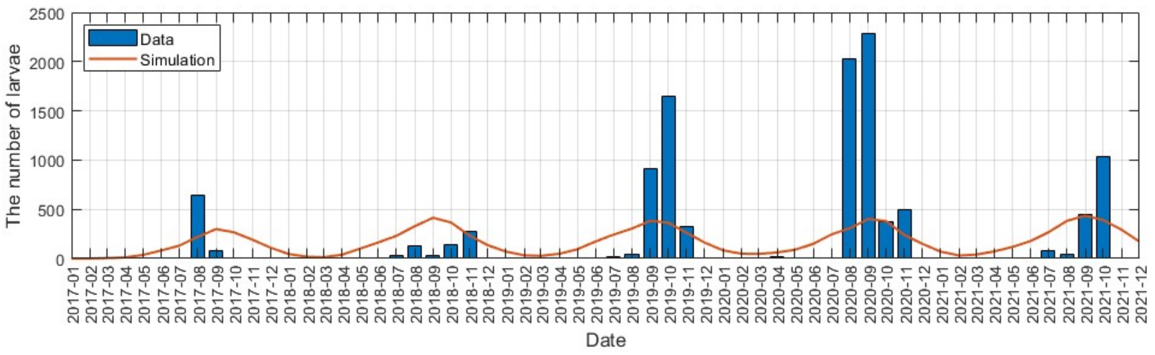

Figure R1 *Collected actual data and estimation of results of incidence of larvae.*

4 More potentially problematics is that the data is not the total number of ticks on the island, or even number per m2 which K is based on, so how do you take that into account in your parameter estimates? Surely is it only question ticks in a particular area? So you need to justify why fitting exactly to that data is the right thing to do. It might have been more appropriate to do the mechanistic modelling and make sure you have the right type of behaviour but not necessarily the right numerical behaviour.

(Answer) Thank you for the comment. While our study does not rely on a comprehensive census of the entire tick population on Jeju Island, it focuses on areas with a high likelihood of human-tick interaction. This approach is justified by the primary transmission route of SFTS, which occurs through tick bites.

Firstly, the carrying capacity, K, was estimated based on the available wood and grass area suitable for tick habitation. This parameterization, while not exhaustive, provides a reasonable approximation of the environmental constraints on tick populations.

Secondly, as stated in lines 112-114, the tick collection methodology targeted specific environments with a high potential for human-tick contact. This strategy ensures that the collected data is relevant to the epidemiological significance of SFTS. While it may not represent the absolute total tick population, it captures the dynamics of the population that poses the greatest risk to human health.

While we acknowledge the limitations inherent in using a localized dataset, we assert that this approach is not only justifiable but strategically advantageous for our research goals. By focusing on the tick population dynamics in areas with high human-tick interaction, our model is better equipped to explore the interplay between climate factors and SFTS transmission risk. The precise fitting to this targeted dataset enhances, rather than diminishes, the model's ability to provide meaningful insights into this important public health issue.

Smaller comments

1 Page 2 line10 delete "year"

(Answer) Thank you for the comment. We have revised it as suggested.

2 Lines 39 to 52 there is a new paper by Worton et al that you could include here (https://royalsocietypublishing.org/doi/full/10.1098/rsif.2024.0004) on modelling ticks with climate change. Also Gilbert L, Aungier J, Tomkins JL . 2014 Climate of origin affects tick (Ixodes ricinus) host-seeking behaviour in response to temperature: implications for resilience to climate change Ecol. Evol. 4 , 1186–1198. (doi:10.1002/ece3.1014) And Gilbert L . 2010 Altitudinal patterns of tick and host abundance: a potential role for climate change in regulating tick-borne diseases?. Oecologia 162 , 217–225. (doi:10.1007/s00442-009-1430-x) Might be of interest for the biology.

(Answer) Thank you for the comment. We have revised the manuscript in lines 45-58 and added more references as recommended.

3 Page 2 line 51 I don't know what a Representative Concentration Pathway is, can you explain briefly?

6 Line 78 again I am afraid I don't know what Shared Socioeconomic pathways scenarios are, is this a standard method? It either needs a reference or more explanation.

10 Page 6 lines 160-164, if these had come earlier then that would have answered some of my previous questions.

(Answer) Thank you for the comments. We answer questions 3, 6, and 10 together. We agree that introducing these concepts earlier in the paper will improve readability and understanding. As recommended, we have moved the sentences explaining RCPs and SSPs from lines 160-164 in the original manuscript to the Introduction (lines 40-45) in the revision.

4 Page 3 line 56 on: I think you should say that Haemaphysalis longicornis is in the Ixodes family and is the main vector of SFTS earlier than you do, so at the start of

the paragraph which starts on page 56 which would make the rest of the paragraph make more sense.

(Answer) Thank you for the comment. As suggested, we have moved the information about *Haemaphysalis longicornis* before the paragraph on line 72 for better context and flow.

5 Line 69 you say shorter development period and higher development rate, are these the same thing or are they more likely to develop ie have a lower death rate while developing? Can you make that clearer here please?

(Answer) Thank you for the comment. A shorter development period and a higher development rate refer to the same concept. Essentially, development rate is calculated as the inverse of the development period. So, a shorter development period automatically means a higher development rate, and vice-versa.

7 Page 4 line 105 your say the data comes from four different environments, but that is not apparent from the graphs, are they all just added together? Do the different environments have different tick abundances? Ie do you use this info about the different environments in your model?

(Answer) Thank you for the comment. The data presented in the graphs represents the combined tick abundance across all four collection environments. While the collections were performed in different environments, the data we analyzed is aggregated. We do not have access to the tick abundance data for each individual location. Therefore, we were unable to incorporate environmental variations in tick abundance into the current model.

We are currently exploring the possibility of obtaining the disaggregated data to address this limitation in future work.

8 Line 116 delete the sentence that starts "additionally" you have already said this in the previous sentence.

(Answer) Thank you for the comment. We have removed the redundant sentence as suggested.

9 Line 120 you refer to larval reproduction, but larvae don't reproduce so what do you mean here?

(Answer) Thank you for the comment. We have replaced "larval reproduction" with "larval development".

11 Page 87 line 196 delete "within"

(Answer) Thank you for the comment. We have revised it as suggested.

12 Line 202 what do you mean by "one month acaricide spraying" . it could mean spraying monthly or just the effect only lasts for a month

(Answer) Thank you for the comment. "one month acaricide spraying" means that the effect of a single acaricide spraying lasts for one month.

13

13.1 Line 207 and 208 I am not sure why you have chosen to write the equations this way. What you have said in the text. I think you say that larve are 57 times more likely than nymphs and 730 times more likely than adults to die of the acaricide (line 204)

So I would say that means CR2*μ_2=730*CR2* μ_4=57*CR2*μ_3, that is sort of what is in the equations, but you have made the nymph death rate the "normal" one and I wondered why, can you at least confirm that the effect parameter was fitted to the nymph data? Perhaps we also need more information about CR2, is this the rate of application of acaricide or just the effect?

(Answer) Thank you for the comment. The control parameter CR_2 represents the effect of one-month acaricide spraying. The derivation and calibration of this parameter are based on findings presented in two references, [59] and [60] in the manuscript (referenced as [1] and [2] below for clarity). Specifically, reference [1] provides experimental data on nymph mortality rates resulting from acaricide exposure. This dataset, focusing on

the nymph stage, served as the foundational basis for parameterizing the one-month acaricide effect.
Reference [2], on the other hand, offers insights into the differential susceptibility to acaricides across various developmental stages of the tick. To integrate the experimental data from [1] into our broader model, which encompasses all life stages, we employed a scaling approach. The mortality rates for developmental stages other than nymphs were estimated by multiplying the nymph mortality rates by stage-specific constants. These constants were derived to reflect the relative sensitivities to the acaricide, as reported in [2]. Consequently, while the nymph mortality data directly informs CR_2, the mortality rates of other stages are expressed relative to this nymphal baseline. In addition, the fitting results of each control parameter, CR_1, CR_2 are provided in Text S1 Section 2.

13.2 If so how does the rate and size of application get included? I think you just have control on and off? Can you say that explicitly here.
(Answer) Thank you for the comment. As described in the section "Estimation of Cost", our model assumes that acaricide application is spatially restricted, focused on areas with high human activity. This assumption is based on current control practices on Jeju Island, as reported by [3], where acaricide application is primarily concentrated along the Olle Trails and at the entrances to tourist sites.
To estimate the area subject to control measures within our model, we calculated the product of the total length of the Olle Trails and an estimated effective width of acaricide application along these paths. This effective width is based on typical road widths in Korea, reflecting the likely reach of acaricide spraying from roadways. We acknowledge that this is a simplification of the actual application process, which may involve variations in the amount of acaricide applied per unit area. However, this approach allows us to approximate the spatial scale of control and its associated costs. A sentence on this area calculation have been newly added to lines 263-266 of the revised manuscript as follows:

"For the cost estimation of control measures, the area subject to control measures is calculated as the product of the total length of the Olle Trail and the effective width of a typical road in Korea, reflecting the likely lateral reach of control measures."

[Reference]

1. Lee DW, Chang KS, Kim MJ, Ahn YJ, Jo HC, Kim SI. Acaricidal activity of commercialized insecticides against Haemaphysalis longicornis (Acari: Ixodidae) nymphs. Journal of Asia-Pacific Entomology. 2015;18(4):715–718.

2. Kim GH, Kim H, Lee HI. Evaluation of the acaricidal efficacy of Haemaphyslis longicornis, a vector of Severe Fever with Thrombocytopenia Syndrome (SFTS). Public Health Weekly Report. 2021;14(43):3040–3049.

3. Jeju 'tick' red light... "Picking a fern" caused the first infectious disease of the year, url: https://www.hani.co.kr/arti/area/jeju/1138569.html

14 Page 10 line 296-300, presumably this is related to figure 3b where d1 and d2 have much bigger changes in response to temp and humidity than d3 and d4 where the y axis has an extremely narrow range?

(Answer) Thank you for the comment. While it might seem intuitive that the wider ranges of d_1 and d_2 in Figure 3b, compared to the narrower ranges of d_3 and d_4, would directly correlate with their higher sensitivity indices, our analysis suggests a more nuanced relationship. The sensitivity analysis results indicate that the magnitude of a parameter's sensitivity index is not solely determined by its range of variation. For instance, while d_3 exhibits a less restricted range in Figure 3b than d_4, its sensitivity index is considerably smaller. Rather than attributing the differences in sensitivity indices primarily to the parameter ranges, it is more accurate to consider the functional role of each parameter within the model.

15 Figure 7, I might add "see tables s2 and S3 to see the tick abundance with no control measures"- that was a question I had when I originally saw the graphs.

(Answer) Thank you for the comment. We agree that explicitly referencing Tables S2 and S3 will enhance the clarity of the results. Accordingly, we have revised the manuscript in lines 324-326 as follows:

"The cumulative number of ticks collected under each control scenario, including scenarios with no control measures implemented for durations of 1M, 2M, 3M, and 4M, are presented in S2 Table and S3 Table, respectively."

16 Figure 8; Did you consider plotting these all on the same scale on the y axis ie so the max value is 10000? It might be helpful for comparison, but if you have tried and it did not work for some reason then ignore this comment!

(Answer) Thank you for the comment. We have modified Figure 8 as suggested:

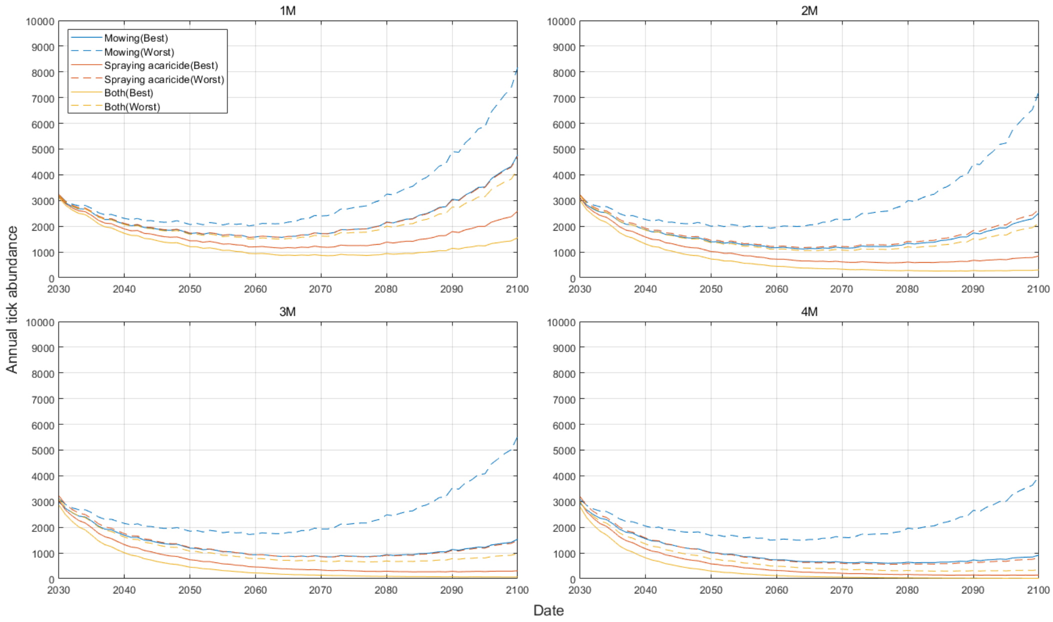

17 Page 11 line 354, I think this is because you are talking about the annual average rather than the cumulative number of patients, but this 5 does not come from the diagram so I found this confusing- can this be seen somewhere? If not perhaps say you "reduce the cumulative number of patients below x which is the equivalent to 5 per year."

(Answer) Thank you for the comment. We have revised corresponding sentence as suggested.

"Furthermore, excluding the scenario with only mowing implemented under SSP5-8.5 with a one-month duration, all other scenarios reduce the cumulative number of SFTS patients below 355, which is equivalent to an average of 5 patients per year."–

18 Figure 9 I don't understand the right hand column, why are there faded and dark bits?

(Answer) Thank you for the comment. In the right-hand column of Figure 9, the shading variations within each color series (blue, red, and green) represent the different components of the total cost. Specifically, the lightest shade corresponds to control costs, the darkest shade represents wage losses, and the intermediate shade indicates medical costs. We have updated the caption of Figure 9 to clarify this distinction.

19 Page 13 line 425. I know this would be complicated to do all possible combinations, but given your results for the single controls did you look what happens if you mow in July and acaricide in May, is that better than doing both in June? I am not requiring you to do this, I was just interested.

(Answer) Thank you for the comment. While our study primarily focused on scenarios where control measures were implemented concurrently or individually, we acknowledge the value of exploring alternative temporal strategies. Our decision to focus on concurrent application was informed by findings from experimental studies conducted in Korea, such as [1], which suggest that mowing is generally less effective than acaricide application and is often considered a supplementary measure. Based on this, [1] recommends implementing both control measures simultaneously to maximize their impact. This recommendation was integrated into the design of our model.

However, we share your interest in the specific scenario of mowing in July and applying acaricide in May. To address this, we have conducted additional simulations, the results of which are presented in Figure R2 below. As illustrated in Figure R2, implementing the control measures in different months results in a smaller reduction in the tick population compared to applying both controls in the same month. These findings further support the recommendation presented in [1], suggesting that the simultaneous implementation of mowing and acaricide application is a more effective strategy for reducing tick populations.

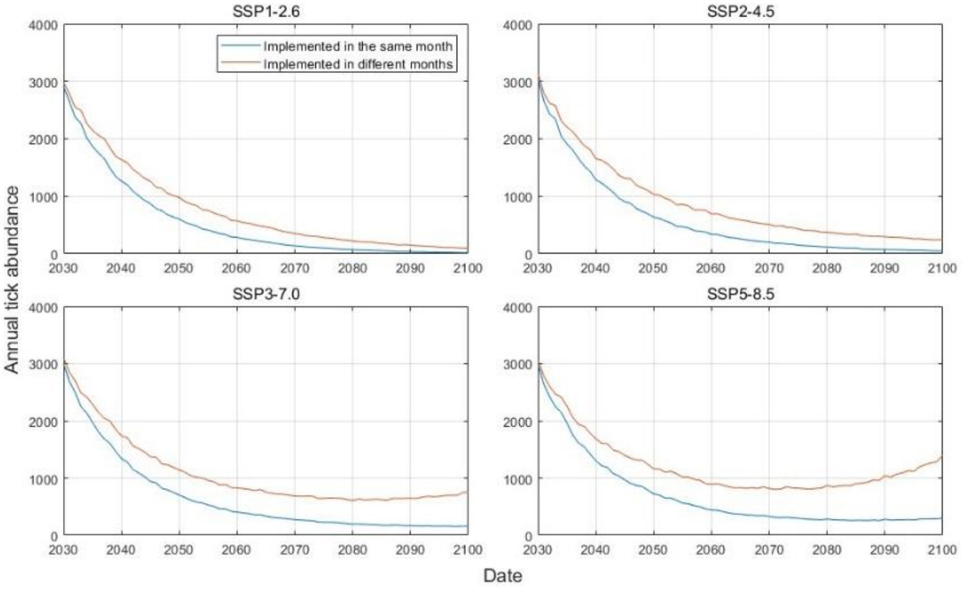

Figure R2 *The annual tick abundance according to each control measure implemented in the same month or in different months (Mowing: July, Spraying acaricide: May)*

[Reference]

1. Chang-Won Jang, Hyunwoo Kim, Gi-Hun Kim, Hee-Il Lee. Evaluation of effectiveness of mowing works for integrated tick control method. Public Health Weekly Report 2021;14:3103-3110.

List of answers to the reviewer 2's comments

We are grateful for the valuable comments from the reviewer and the opportunity to generate a revised manuscript. In this revision, we addressed all of the reviewer's comments and have made an effort to better explain the relevance of our results.

Overall Comment

The authors develop an ODE system to describe the dynamics of a tick population with reproduction and transitions depending on both temperature and humidity. The model is used to predict long term tick abundances, disease risk, and associated costs under different climate change scenarios and control strategies. The specific application considered in this paper is for Severe Fever with Thrombocytopenia Syndrome on Jeju Island in South Korea.

Point by point response

Model formulation

1. The choice for density dependence needs further explanation/justification. Since d1 is the transition rate from eggs to adults, this term seems to imply that the hatching rate (either success in hatching and/or length of incubation) is density dependent. Do you have evidence for this? Moreover, this choice of nonlinearity does not appear to preserve non-negativity of the system since dL/dt |_L = 0 may be negative.
   (Answer) Thank you for the comment. The density-dependent term in the equation for dL/dt is indeed intended to capture the impact of population density on the hatching rate. While direct experimental evidence for this specific relationship in the context of our model may be limited, there is substantial ecological literature such as [1] supporting the notion that population density can influence various life history traits, including hatching rates.

Regarding the potential for negative values of dL/dt at $L = 0$, the carrying capacity, K, in our model is calculated based on the total available habitat on Jeju Island as follows:

$$K = M \times (A_W \times C_w + A_G \times C_G)$$

Parameter	Definition	Value	Reference
M	Maximum number of ticks per host	200	[2]
A_W	Woods area in Jeju Island (m^2)	847180000	[3]
C_w	Carrying capacity for host per m^2 in wood area	0.002	[2]
A_G	Grass area in Jeju Island (m^2)	154550000	[4]
C_G	Carrying capacity for host per m^2 in grass area	0.001	[2]

*Table R2 Parameter description for the carrying capacity (**this part has been newly added in Table 1 of the main manuscript.)***

The value of K significantly exceeds the maximum projected vector population under any of the climate change scenarios (SSPs). This is illustrated in Figure R1 below. As a result, the term $(1 - N_V/K)$ remains positive throughout our simulations, ensuring the non-negativity of dL/dt.

Additionally, it's worth noting that several studies in the field of ecological modeling have adopted similar approaches, where the carrying capacity is set to a large value to prevent unrealistic population fluctuations [5,6,7,8]. In [9], the carrying capacity of vectors was estimated based on various factors, including habitat suitability.

To further emphasize the importance of incorporating carrying capacity, we present Figure R2, which depicts the simulated tick trends without considering density dependence. As evident from the figure, the tick population grows exponentially, leading to an unrealistic scenario. This highlights the crucial role of carrying capacity in generating more realistic model predictions.

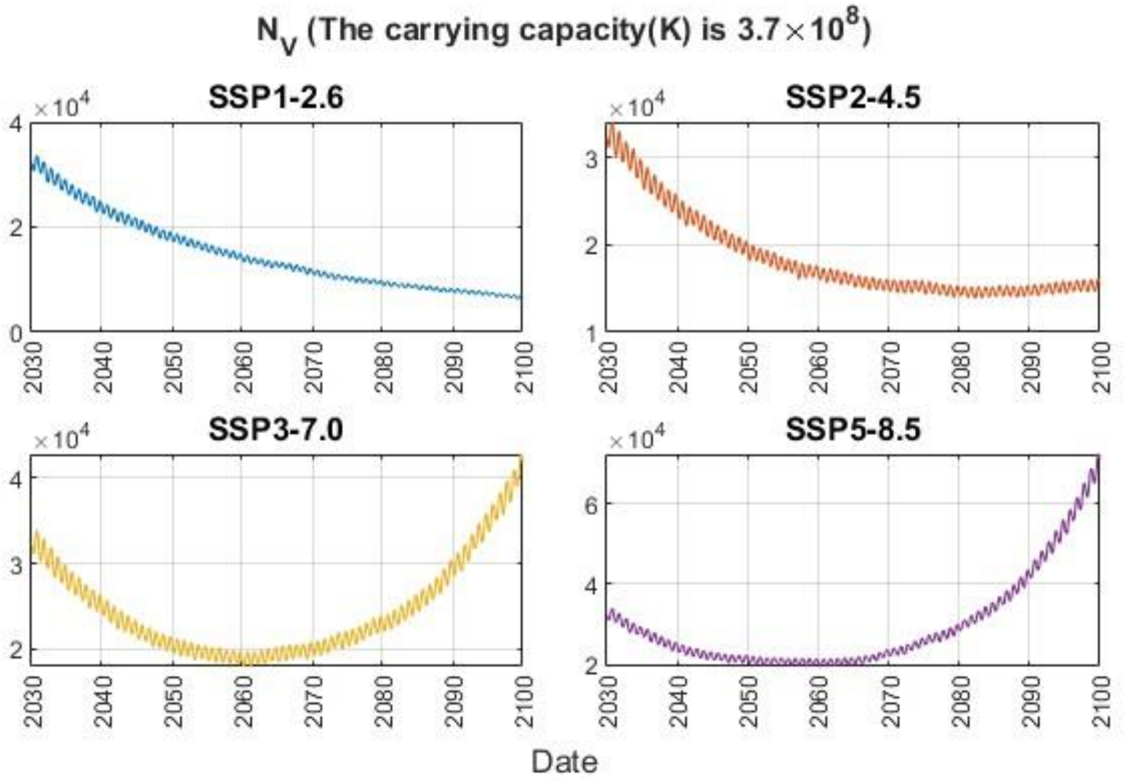

Figure R1 *N_V according to the climate change scenarios.*

–

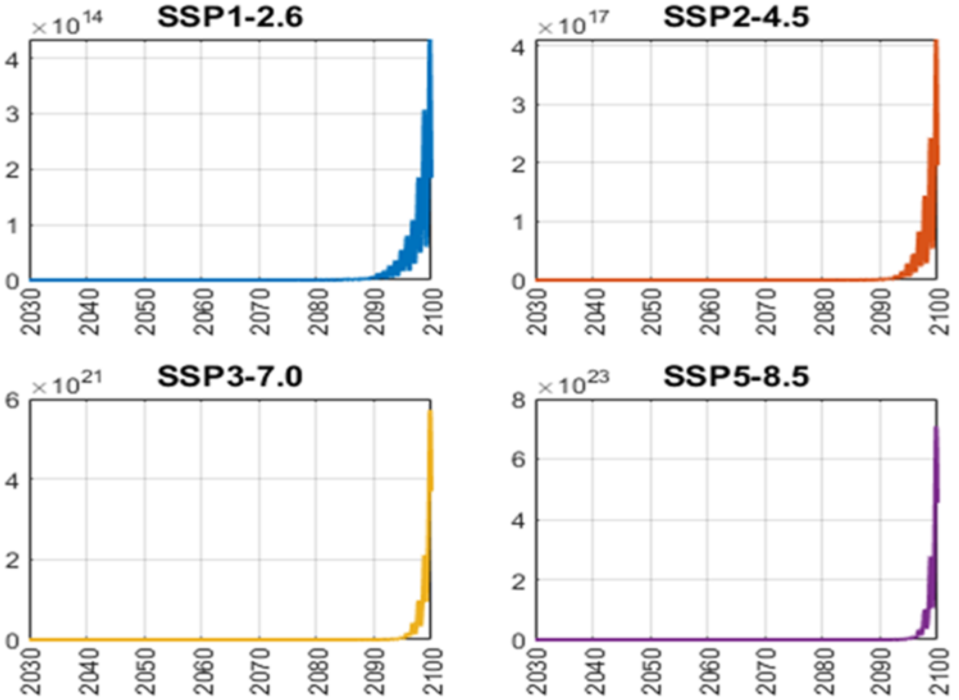

Figure R2 *The monthly abundance of ticks from 2030 to 2100 according to the SSP scenarios without carrying capacity.*

[Reference]

1. Kada, Sarah, Karen D. McCoy, and Thierry Boulinier. "Impact of life stage-dependent dispersal on the colonization dynamics of host patches by ticks and tick-borne infectious agents." *Parasites & Vectors* 10 (2017): 1-12

2. Gaff, Holly D., and Louis J. Gross. "Modeling tick-borne disease: a metapopulation model." *Bulletin of Mathematical Biology* 69 (2007): 265-288.

3. Jeju City Civil Service Office. Forests and fields area generalization (unit: Ha);

2022. https://www.jejusi.go.kr/information/open/data/view.do?id=447.

4. Kim S. There is an urgent need for measures to reduce grassland in Jeju every

year due to various development projects.; 2023.

https://www.jejunews.com/news/articleView.html?idxno=2199710.

5. Traoré, Bakary, Ousmane Koutou, and Boureima Sangaré. "A global mathematical model of malaria transmission dynamics with structured mosquito population and temperature variations." *Nonlinear Analysis: Real World Applications* 53 (2020): 103081.

6. Lee, Eva K., Yifan Liu, and Ferdinand H. Pietz. "A compartmental model for Zika virus with dynamic human and vector populations." *AMIA Annual Symposium Proceedings*. Vol. 2016. 2017.

7. Ngonghala, Calistus N., Miranda I. Teboh-Ewungkem, and Gideon A. Ngwa. "Observance of period-doubling bifurcation and chaos in an autonomous ODE model for malaria with vector demography." *Theoretical ecology* 9 (2016): 337-351.

8. Ngwa, Gideon A., et al. "On a reproductive stage-structured model for the population dynamics of the malaria vector." *Bulletin of mathematical biology* 76 (2014): 2476-2516.

9. Song, Yateng, et al. "Mathematical model analysis and simulation of visceral leishmaniasis, Kashgar, Xinjiang, 2004–2016." *Complexity* 2020.1 (2020): 5049825.

2. In addition, it is incorrect to say “larval reproduction” since larva do not reproduce (pg 4, line 121).

(Answer) Thank you for the comment. We have revised "larval reproduction" to "larval development".

3. In model (1) when defining AF and AM, use alpha and 1-alpha, respectively, for clarity. While your model is technically correct as written since you estimate alpha = ½, for a different estimate this would be incorrect.

   (Answer) Thank you for the comment. We have revised the system of ordinary differential equations (1) and (3) as suggested. Also, Figure 2 has been updated accordingly to reflect these changes.

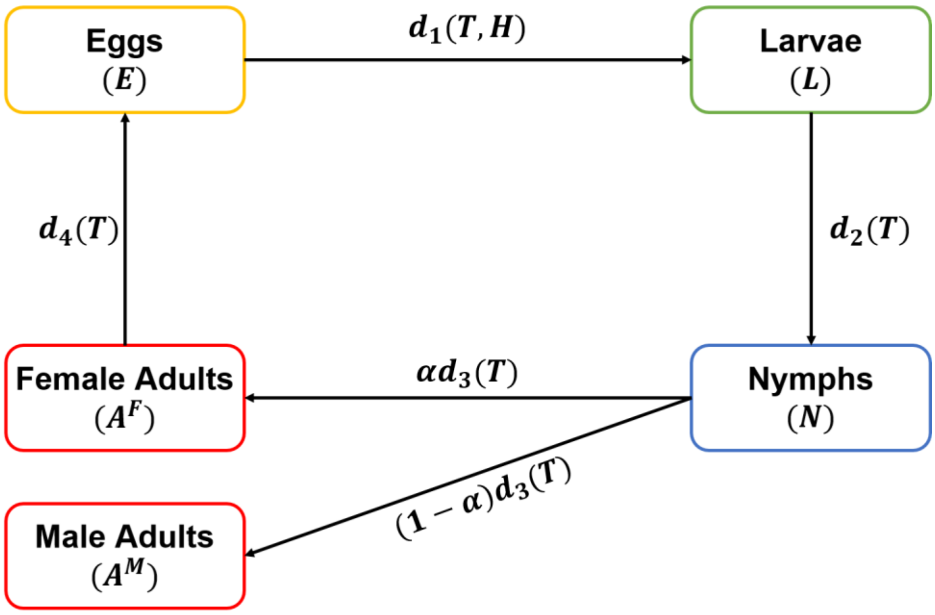

4. Equation (4): For many hard tick species, adult male ticks do not take a bloodmeal. Is this the case here? Please clarify whether the total number of SFTS patients depends on total number of adults or just the number of female adults.

   (Answer) Thank you for the comment. Unlike some hard tick species, both male and female adults of *Haemaphysalis longicornis*, the focal vector in this study, require bloodmeals [1,2]. Therefore, our model considers all adult ticks, both males and females, capable of feeding and potentially transmitting the SFTS virus. Consequently, the incidence of SFTS is dependent on the total number of adult ticks, encompassing both female and male adults.

[Reference]

1. MATSUO, Tomohide, Takayuki MORI, and Satoshi SHIRAISHI. "Histological and histochemical studies on the male accessory genital glands and the derivations of the spermatophore components from the accessory glands in Haemaphysalis longicornis (Acari: Ixodidae)." ***Journal of the Acarological Society of Japan*** **6.1 (1997): 1-10.**

2. Matsuo, Tomohide, and Takayuki Mo° ri. "The significance of feeding for reproduction in a male Metastriata tick, Haemaphysalis longicornis (Acari: Ixodidae)." ***Acta Zoologica*** **81.1 (2000): 17-26.**

5. Related: Pg 9, lines 300-301: Is it almost zero or is it zero? If the adult males do not take bloodmeals, then I don't see how males would impact this calculation at all.
(Answer) Thank you for the comment. The sensitivity index for adult male mortality is not exactly zero, but rather very close to zero. In Fig. 6, the values for μ_5 appear to be zero due to scale differences with other parameters. However, when we plot μ_5 independently, as shown in Figure R3 below, we can see that the values of μ_5 are not zero. Figure R3 shows that the sensitivity index of the cumulative number of ticks about μ_5 under the SSP scenarios. Furthermore, as previously stated, adult males of *Haemaphysalis longicornis* do require bloodmeals. However, the sensitivity analysis, which was based on the cumulative number of ticks, reveals that changes in the mortality of adult males have a minimal effect on total tick abundance. This indicates that, while male ticks contribute to the population, variations in their mortality rate have a relatively minor effect on the overall tick population size in our model, likely due to other factors having a more dominant influence.

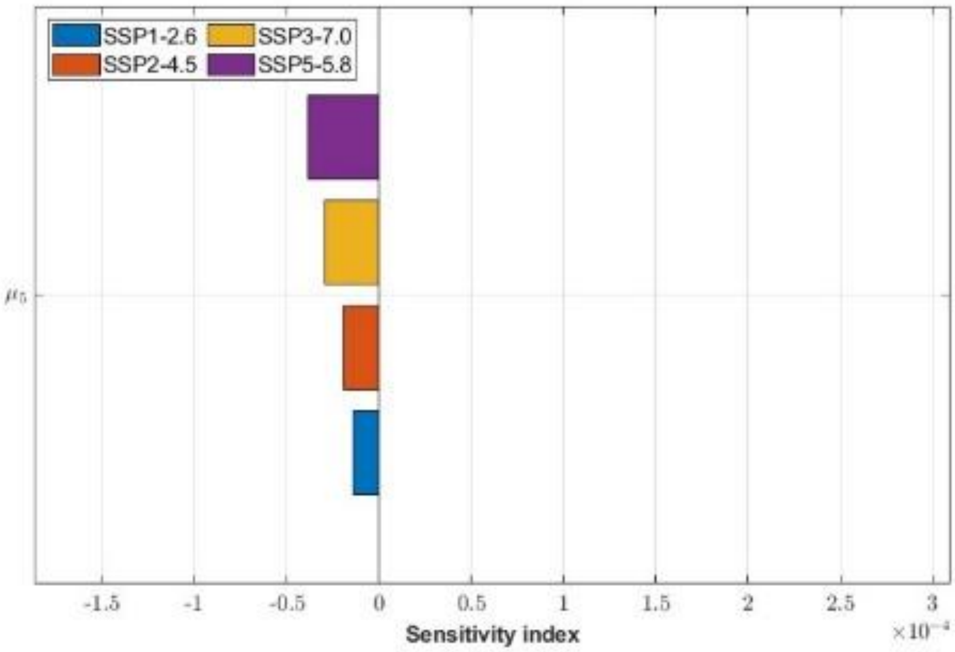

Figure R3 *Sensitivity index of the cumulative number of ticks about* μ_5 *under the SSP scenarios. The specific values of each sensitivity indices are -1.44e-05, -1.96e-05, -2.93e-05, and -3.82e-05 for SSP1-2.6, SSP2-4.5, SSP3-7.0, and SSP5-8.5, respectively.*

6. Study site and control measures: Please include some measure of scale here. What is the spatial scale under consideration in this study? Is it the entirety of Jeju Island? And if yes, does, for example, mowing assume that mowing occurs over the entire area.

   (Answer) Thank you for the comment. As described in the section "Estimation of Cost", our model assumes that acaricide application is spatially restricted, focused on areas with high human activity. This assumption is based on current control practices on Jeju Island, as reported by [1], where control measures are primarily concentrated along the Olle Trails and at the entrances to tourist sites.

   To estimate the area subject to control measures within our model, we calculated the product of the total length of the Olle Trails and an estimated effective width of control measures along these paths. This effective width is based on typical road widths in Korea, reflecting the likely reach of control measures from roadways.

   A sentence on this area calculation have been newly added to lines 263-266 of the revised manuscript as follows:

"For the cost estimation of control measures, the area subject to control measures is calculated as the product of the total length of the Olle Trail and the effective width of a typical road in Korea, reflecting the likely lateral reach of control measures."

[Reference]

1. Jeju 'tick' red light... "Picking a fern" caused the first infectious disease of the year, url: https://www.hani.co.kr/arti/area/jeju/1138569.html

Results

7. Figures: Some of the figures (e.g., Fig 5, 8) are missing axes labels.

(Answer) Thank you for the comment. We have revised the figures as suggested:

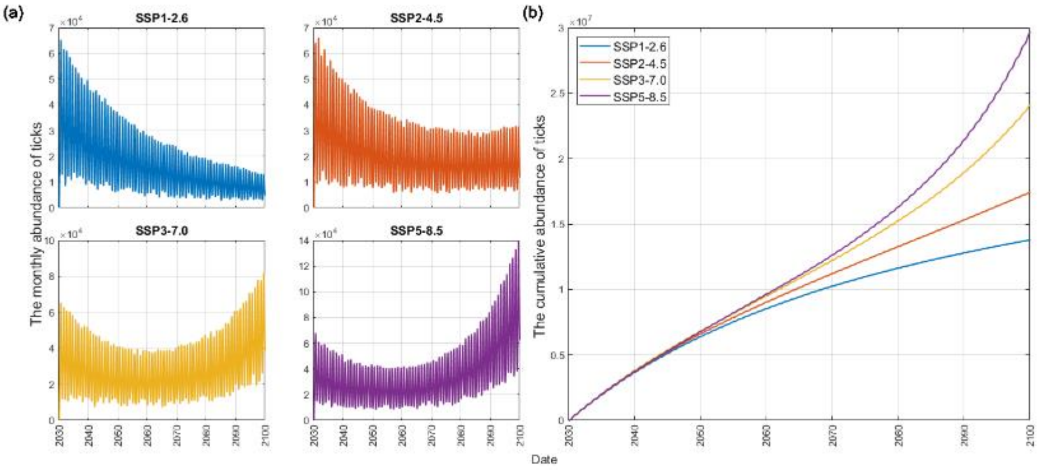

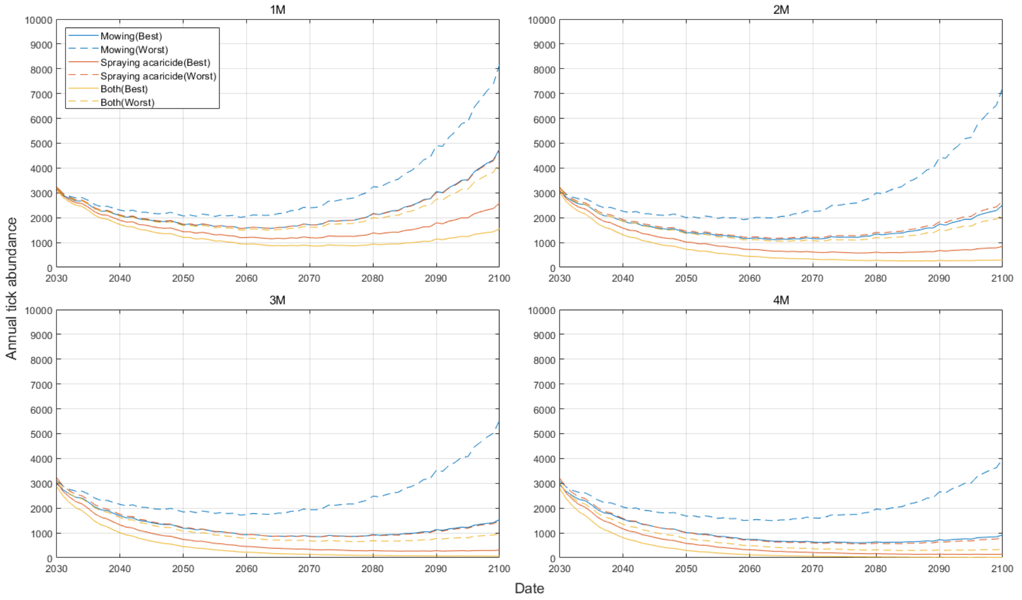

8. Figure 5: Can you provide an explanation as to why in many of the scenarios we observe an initial decrease in the tick population?

(Answer) Thank you for the comment. The initial decrease in tick population observed in many scenarios can be attributed to the difference in temperature between the baseline year (2021) and the initial years of the projected period. Our model uses 2021 temperature data as a baseline for the period from 2021 to 2030. However, a comparison of the 2021 temperatures with the actual measured temperatures for 2022 and 2023 reveals that 2021 experienced higher temperatures in most months. This implies that the baseline temperature used for the initial years of the simulation is higher than the actual temperatures experienced in those years.

Since tick population dynamics are highly sensitive to temperature, this discrepancy in temperature leads to an initial decrease in the tick population. The model, calibrated to 2021 temperatures, predicts a higher tick population in the initial years. However, when faced with the lower actual temperatures of 2022 and 2023, the simulated population declines to reflect the less favorable conditions. This phenomenon is further emphasized when considering the SSP1-2.6 scenario from 2030 to 2032. Comparing the SSP1-2.6 temperature data with the 2021 data shows that 2021 temperatures are significantly higher (by almost 4°C) in most months, except for July and August. Therefore, when the simulation transitions to the SSP1-2.6 scenario with its lower temperatures, a decline in the already increased tick population is observed.

For a more detailed discussion on this phenomenon, please refer to Text S2 in Supporting Information.

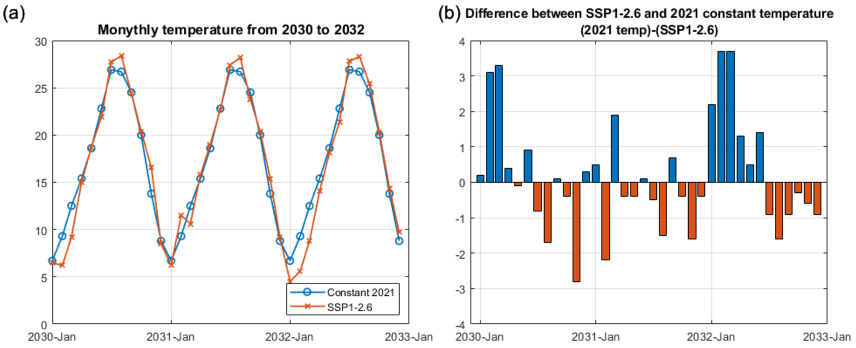

Figure R4 *Comparison of the 2021 actual temperature and SSP1-2.6 average temperature from 2030 to 2032. (a) Compare the 2021 temperature and projection of SSP1-2.6 monthly temperature from 2030 to 2032 (b) Difference between 2021 temperature and SSP1-2.6 from 2030 to 2032*

9. Figure 7 caption: Remove “collected”. (I am assuming this is actual tick counts from the model, not a sampling estimate.)

   (Answer) Thank you for the comment. We have revised it as suggested.

10. Figure 9: I had difficulty reading the cost graph due to the resolution, but if I am reading this correctly, it looks like the control costs are significantly higher than the medical and wage loss costs. Given the information you have available, does this seem accurate?

    (Answer) Thank you for the comment. In the right-hand column of Figure 9, the shading variations within each color series (blue, red, and green) represent the different components of the total cost. Specifically, the lightest shade corresponds to control costs, the darkest shade represents wage losses, and the intermediate shade indicates medical costs. We have updated the caption of Figure 9 to clarify this distinction.

    Your observation that control costs constitute the largest proportion of the total cost is correct. Although this may seem surprising, it is important to consider the broader context provided by the cost-benefit analysis detailed in Text S3 in Supporting Information. This analysis reveals that, despite the relatively high cost of control measures, their implementation consistently yields a cost benefit when compared to the costs associated with inaction, which include medical expenses and productivity losses due to SFTS.

Discussion

11. I think it may be worth pointing out in the discussion that, while the modeling of the tick population dynamics is detailed, the modeling of pathogen dynamics is very simplified. Specifically, disease risk is based on tick abundance and does not, for example, explicitly include dependence on competent hosts or pathogen dynamics. For example, it has been found that larva survival plays an important role in R0 for certain tick-borne diseases (e..g, Ostfeld Richard S. and Brunner Jesse L. 2015 Climate change and Ixodes tick-borne diseases of humans Phil. Trans. R. Soc. B37020140051).
    (Answer) Thank you for the comment. We acknowledge this as a valid limitation of our study and have revised the discussion accordingly in lines 472-479 in Discussion.

    "Fourth, while we incorporate the influence of climate change on tick abundance, we acknowledge that climatic factors can also impact pathogen dynamics directly, as well as indirectly through effects on the density and behavior of reservoir hosts critical for pathogen maintenance. For instance, Ostfeld and Brunner highlighted the significant role of larval survival, influenced by climate, for certain tick-borne diseases [1]. Our current model does not explicitly account for these intricate host-pathogen interactions, focusing primarily on the broader relationship between climate change and tick population dynamics."

    [Reference]

    1. Ostfeld, Richard S., and Jesse L. Brunner. "Climate change and Ixodes tick-borne diseases of humans." ***Philosophical Transactions of the Royal Society B: Biological Sciences*** **370.1665 (2015): 20140051.**

Minor points

1. Remove “the” before “elderly” in the abstract

   (Answer) Thank you for the comment. We have revised it as suggested.

2. Pg 2, line 7: “range” ◊ “ranges”

   (Answer) Thank you for the comment. We have revised it as suggested.

3. Pg 2, line 10: remove “year”

   (Answer) Thank you for the comment. We have revised it as suggested.

4. The models (equations (1) and (3)) should be given in a sentence

   (Answer) Thank you for the comment. We have revised it as suggested in line 144 and line 223.

5. Pg 13, line 417 add “the” between “reduce” and “tick”

   (Answer) Thank you for the comment. We have revised it as suggested.
